# A survey of the working status of family medicine physicians in clinics and hospitals in Korea

**DOI:** 10.1186/s12875-020-01154-5

**Published:** 2020-05-08

**Authors:** Hyun-Young Shin

**Affiliations:** Department of Family Medicine, College of Medicine, Myongji Hospital, Hanyang university, 697-24 Hwajung-dong, Deokyang-gu, Gyeonggi-do, 412-270 South Korea

**Keywords:** Continuity of care, Family medicine, Practice management, Primary care

## Abstract

**Background:**

In the approximately 35 years since family medicine was established in South Korea, family medicine physicians have sought to expand their expertise to cover clinical fields beyond primary medicine. This study examines their working status and compares the working conditions of family medicine physicians in clinics and hospitals in Korea.

**Methods:**

We conducted an online survey with 4057 family medicine physicians in Korea in 2016. The results were analyzed using descriptive statistics.

**Results:**

Of the respondents, 572 doctors were working in clinics and 441 in hospitals. In the analysis of treatment pattern by doctors, the rate of chronic disease management was 84.7% in clinics and 93.4% in hospitals (*p* <  0.001), and the rate of diseases covered by national insurance was 74.8% in clinics and 76.9% in hospitals (*p* = 0.005). Among physicians younger than 40 years, the rate of chronic disease management and diseases covered by national insurance were 64.6 and 68.0% in clinics and 93.6 and 78.5% in hospitals, retrospectively.

**Conclusions:**

Family medicine physicians working in hospitals have higher rates of chronic disease management and diseases covered by national insurance. This discrepancy of treatment pattern became larger for doctors younger than 40 years. More in-depth studies of the treatment pattern and its tendencies between family medicine physicians in clinics and hospitals are needed in the future.

## Background

Since family medicine from US was introduced in Korean medical system in 1978, the first family medicine resident program was started in 1979. The Korean Academy of Family Medicine was established in 1980 and it became the one of the official members in WONCA (World Organization of National Colleges, Academies and Academic Associations of General Practitioners/Family Physicians) in 1983. Family medicine was designated as the 23rd specialty board in 1986 in Korea, and 8024 family medicine specialists had been certified as of 2016 with 135 training hospitals and 900 family medicine residents [1]. Since then, the Family Medicine Society has been making efforts in various fields to be “the center of primary care in Korea” [1]. However, as a result of the specialization and subdivision of western medicine, instability of the medical delivery system, and other political problems, family medicine physicians in Korea have expanded their practice to include not only comprehensive primary health care but also a variety of other fields, including disease prevention and health promotion such as health check-up centers, clinics providing cosmetic medical services, anti-obesity clinics, and functional medicine in areas not covered by government insurance [2].

Specialists make up 75.9% of Korean doctors, and 92.6% of clinic doctors have diversity specialist boards [3–5]. Because of competition with other clinics and the absence of insurance coverage ensuring continuity, comprehensiveness, and family care in the medical system, the function of primary medicine as a gatekeeper has been weakened. Furthermore, patients prefer to visit secondary and tertiary medical institutions as limitations to access to large hospitals are rare, which has brought about an unstable medical delivery system in Korea [6]. Although about more than 35 years have passed since family medicine departments were established in Korea, there have been no reports about family medicine physicians’ working status. This study examines the current working status of family medicine physicians and compares the working conditions of physicians working in clinics and hospitals.

## Methods

### Study population

**Fig. 1:**
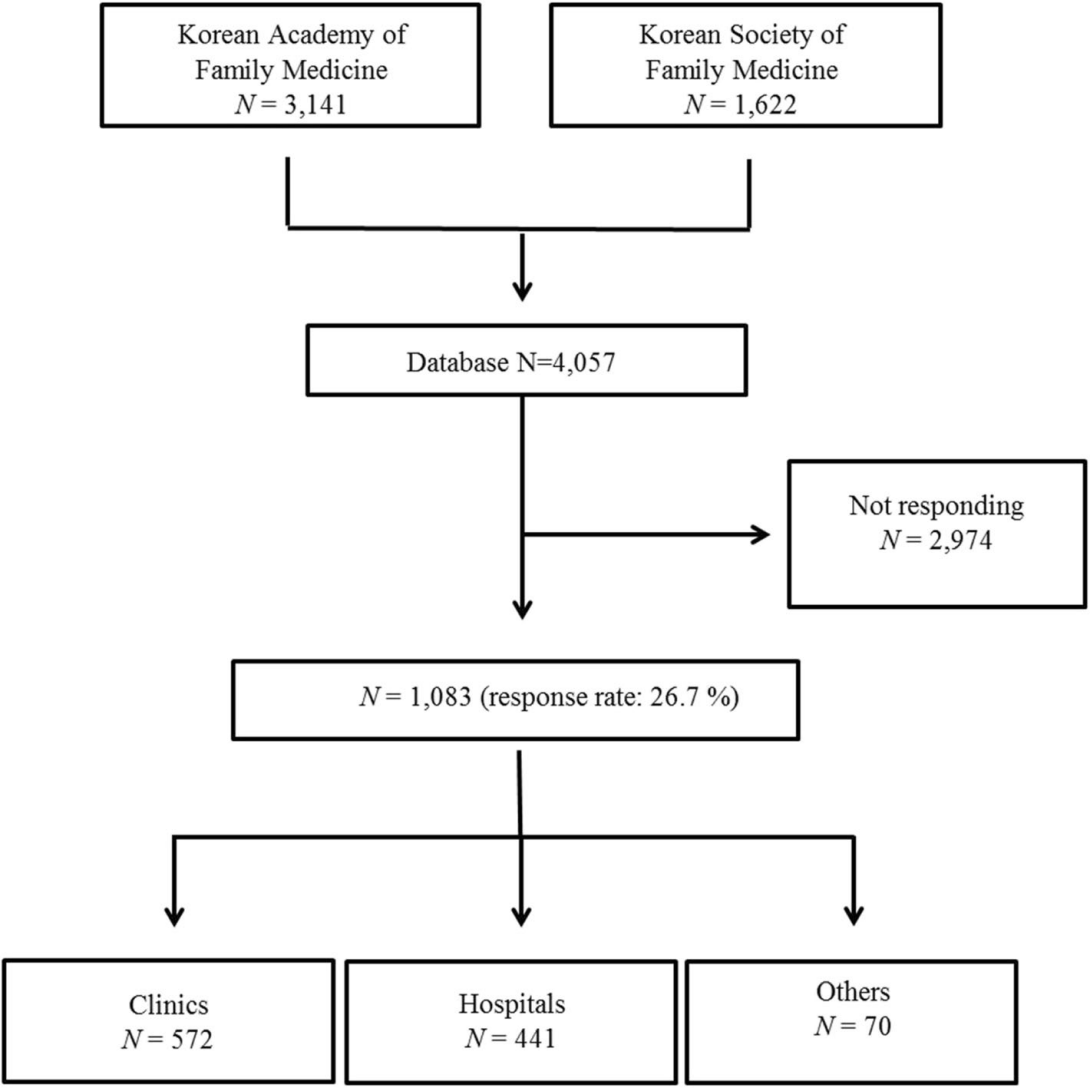
Flow chart of study population of the present study (2016)

A list of family medicine physicians was obtained through the databases of the Korean Academy of Family Medicine (3141 persons) and the Korean Society of Family Medicine (1622 persons) (Fig. 1). Members’ e-mail addresses and text message contact information were provided by the database holders after confidentiality was assured. All participants provided written informed consent, and this study was approved by the Myongji Hospital Institutional Review Board (MJH-16-097).

### Questionnaire development and survey process

The questionnaire elicited the respondents’ basic information, including age and sex, board duration, education level (bachelor, master, doctor), region of medical institution (large city, small-medium city, rural area), their workplace (clinic, hospital, geriatric hospital, general hospital, advanced general hospital), and position in the medical institution (clinic: owner, paid, others; hospital: professor, resident, paid, others). Two major groups were classified as ‘clinic’ group was the doctors working in clinic and ‘hospital’ group was the doctors working in hospital, geriatric hospital, general hospital, and advanced general hospital. In Korea, doctors are usually working on one medical institute, which means practice in clinics and hospitals are mutually exclusive. Chronic disease management was assessed by the question, “Have you provided treatment of chronic disease such as diabetes and hypertension for patients?” (answered *yes* or *no*); diseases covered by national insurance by “Indicate the percentage of your patients covered by national health insurance”; and patient age groups by “What proportions of your patients are younger than 18, aged 19–64, and older than 65?” For working conditions, working days per week and working nights per week were asked, with the night shift deemed as starting from 18:30. Online surveys were conducted for about 3 months from August 15 to December 15, 2016. Participants were regularly e-mailed and sent text messages to encourage participation.

### Data analysis

Descriptive statistical analyses were performed on the results of the survey. The mean value and standard deviation of the continuous variables and the median and standard deviation of the categorical variables were calculated. The statistical analysis of the two groups (working in clinics and hospitals) was conducted with Student’s *t*-tests and chi-square tests. Statistical significance was defined as *p* <  0.05. SAS statistical software, version 9.4 (SAS Institute Inc., Cary, NC), was used in this study.

## Results

**Fig. 2:**
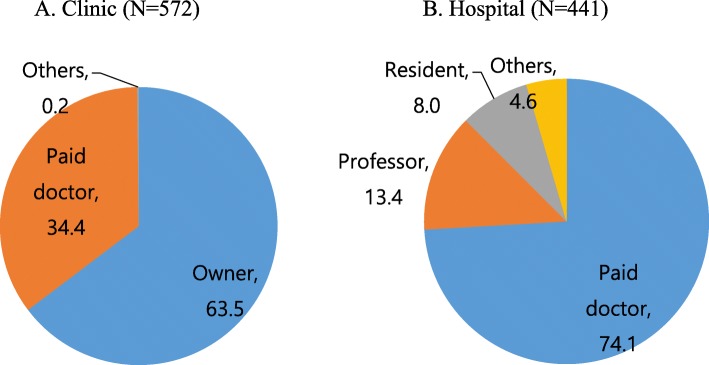
Distribution of positions of family medicine physicians in Korea**.** 2A. Clinic (*N* = 572). 2B. Hospital (*N* = 441)

Among the total 4057 family specialists, 1083 potential respondents answered for a response rate of 26.7%. Of these, 572 worked in clinics and 441 in hospitals (Fig. 1). Table 1 shows the clinical characteristics of the family medicine physicians working in clinics and hospitals. The mean age was higher in clinics than in hospitals (43.7 vs. 41.2 years old, *p* <  0.001, respectively), the proportion of women was lower in clinics than in hospitals, and the duration since board certification was longer in clinics than in hospitals (27.6 vs 38.6%, *p* <  0.001, 10.8 vs 8.6 years, *p* <  0.001, respectively). Regarding the classification of the medical institutions where doctors are working, 16.6% worked at advanced general hospitals, 28.6% at general hospitals, 32.4% at geriatric hospitals, and 22.4% at hospitals. Figure 2 shows the distribution of family medicine physicians in Korea by employment status; doctors employed in and owning clinics made up 34.4 and 63.5%, respectively, while among the doctors working in hospitals, 74.1% were paid doctors, 13.4% professors, 8.0% residents, and 4.6% others.

**Table 1:** Clinical characteristics of the family medicine physicians working in clinics and hospitals (2016)

Variables	Clinic ***N*** = 572	Hospital ***N*** = 441	*p value
Age (years)	43.7 ± 8.7	41.2 ± 8.8	< 0.001
Women (number/%)	158 (27.6)	170 (38.6)	< 0.001
Board duration (year)	10.8 ± 8.0	8.6 ± 7.9	< 0.001
Education level (number/%)			< 0.001
Bachelor	408 (71.5)	227 (51.9)	
Master	130 (22.8)	128 (29.3)	
Doctor	33 (5.8)	82 (18.8)	
Region of medical institution (number/%)			0.08
Large city	338 (59.3)	230 (52.9)	
Small-medium city	184 (32.6)	176 (40.5)	
Rural area	42 (7.4)	29 (6.7)	
Classification of medical institution (number/%)			< 0.001
Advanced general hospital		73 (16.6)	
General hospital		126 (28.6)	
Geriatric hospital		143 (32.4)	
Hospital		99 (22.4)	
Clinic	572 (100)		

All data are represented as mean ± standard deviation or number(%)

*p value from Student’s *t*-test or chi-square test

Table 2 shows the results for medical practice characteristics provided by family medicine physicians working in clinics and hospitals. First, the rate of chronic disease management was 84.7% in clinics and 93.4% in hospitals (*p* <  0.001). Among the physicians younger than 40 years old, the rate of chronic disease management was 64.6% in clinics and 93.6% in hospitals (*p* <  0.001), while among those 40 years old and older, the rate of chronic disease management was 93.6% in clinics and 92.7% in hospitals (*p* = 0.31). The rate of diseases covered by national insurance was 74.8% in clinics and 76.9% in hospitals (*p* = 0.005). Among the physicians younger than 40 years old, the rate of disease covered by national insurance was 68.0% in clinics and 78.5% in hospitals (*p* <  0.001), while among physicians 40 years old and older, the rate of disease covered by national insurance was 81.0% in clinics and 84.7% in hospitals (*p* = 0.19). Regarding patients’ age groups, the percentages younger than 18, aged 19–64, and 65 and older were 14.5, 54.8, and 31.1% in clinics and 6.7, 37.7, and 55.6% in hospitals, respectively (*p* <  0.001).

**Table 2:** Medical characteristics provided by family medicine physicians working in clinics and hospitals (2016)

		Clinic ***N*** = 572	Hospital ***N*** = 441	*p value
Chronic disease management		84.7%	93.4%	< 0.001
Younger than 40 years old		64.6%	93.6%	< 0.001
40 years old or older		93.6%	92.7%	0.31
Disease covered by national insurance		74.8%	76.9%	0.01
Younger than 40 years old		68.0%	78.5%	< 0.001
40 years old or older		81.0%	84.7%	0.19
Patients’ age group (years)	< 18	14.5	6.7	< 0.001
	19–64	54.8	37.7	
	≥ 65	31.1	55.6	

*p value from Student’s *t*-test or chi-square test

Table 3 shows the results for the working conditions of family medicine physicians during the day and the night per week. Mean working days per week were 5.7 and 5.3 in clinics and hospitals, respectively (*p* <  0.001), and mean working nights per week were 3.0 and 0.8 in clinics and hospitals, respectively (*p* <  0.001). Supplementary Figure 1 presents the distribution of working days per week and working nights per week. Family medicine physicians’ working days most commonly worked 6 days a week in clinics and 5 days a week in hospitals, while those working nights most commonly worked 5 days a week and second most commonly zero days in clinics, and zero days a week in hospitals.

**Table 3:** Working conditions of family medicine physicians, days and nights per week (2016)

Mean working days (day/week)	Clinic	Hospital	***p*** value*
Day	5.7 ± 0.8	5.3 ± 0.7	< 0.001
Night	3.0 ± 2.3	0.8 ± 1.3	< 0.001

* Student’s *t*-test between clinic and hospital workers; Night shift starts from 18:30

1A. Working days per week

1B. Working nights per week

## Discussion

In this study, we found that family medicine physicians working in hospitals have higher rates of chronic disease management and higher rates of elderly patients than those working in clinics, and this discrepancy became larger in doctors younger than 40 years.

There are several possible reasons for the differences in medical treatment patterns of family medicine physicians between hospitals and clinics. Family medicine physicians in Korea have expanded to provide medical services not only for disease treatment but also health check-up centers, health promotion medical services, and new advanced medical technologies, which are classified as medical areas not covered by national insurance [7]. As the proportion of total medical expenses classified as procedures not covered by national insurance has increased steadily in Korea, this phenomenon has also extended to family medicine physicians’ practice patterns [8, 9]. According to a report by the National Health Insurance Service (NHIS), medical expenses not covered by national insurance doubled during 2009 to 2014, and the proportion of noncovered health insurance expenses gradually increased from 13.4% in 2006 to 17.1% in 2014 [10]. Moreover, the number of clinicians practicing only in noncovered medical areas in general medicine, plastic surgery, dentistry, and other fields has doubled in the last 5 years [9]. It is recognized that the appearance-oriented culture that emphasizes measures such as anti-obesity treatments is one of the factors contributing to the demand for the various cosmetic procedures, including skin care treatments, plastic surgery, and anti-aging treatments [11, 12]. In this survey, 44.8% of family medicine physicians answered that the reasons for choosing a noncovered medical area are economic, 20.1% personal interest, and 10.4% to achieve independence from government restrictions on the right to medical treatment (Supplementary Table 1). There is a similar report concerning operating a clinic for economic reasons and the considerations affecting the choice of treatment area from the annual report of Korean Medical Association, which means that this phenomenon extends beyond family medicine physicians to other specialists who operate clinics [4].

Another reason for the changing medical practice patterns in young family medicine doctors is the instability of the medical delivery system, which has reduced the influence of primary medical institutions in the medical market share [13]. The concept of primary care with a gatekeeper has not been established yet, and there are still many challenges to address before the possibility of each patient having his or her own primary physician becomes a reality in the Korean medical system [14, 15]. The number of family physicians who are newly entering the medical market has increased and clinicians, who are more vulnerable to the deterioration of the medical delivery system, may have been careful about incurring economic problems when choosing their primary care area, which is an issue more pressing for family medicine physicians working in clinics than in hospitals [13]. For these reasons, we may assume that the trends in the medical treatment provided by young doctors are more likely to result in providing health promotion medical services than chronic disease management. More in-depth study of the differences in the tendencies of family medicine doctors working in clinics and hospitals are needed in the future.

This study also found that the number of days spent working days and nights were significantly higher for doctors working in clinics than in hospitals. This might be related to the operating hours of clinics and hospitals, and probably one of the reasons is that clinics usually open later and close later than hospitals. In addition, this factor is strongly affected by the medical treatment area and practice pattern in hospitals; for example, if a family medicine department in a hospital is oriented to outpatient medical care and health checkup centers, then there is no need for inpatient care and night duty work. In contrast, if a hospital is centered on hospice-palliative inpatient care, this requires more night duty work by family medicine doctors. A more detailed evaluation of working hours should be performed in the future for a more detailed assessment of doctors’ work environments.

The limitations of this study are as follows. First, the questionnaire survey is limited by subjective factors of respondents, possibly leading to under-reporting or over-reporting. Second, this study was conducted on the subjects who agreed with the usage of the personal information, and the limitation of the update of the group database in the society. Therefore, the representativeness of the samples was limited because our study was conducted in half of the total family physicians in Korea and the number of respondents was not enough. Third, the study is limited by the lack of information on population samples covering the whole range of family medicine specialists and the standardization of the participants in terms of sex, age, and regional area. Therefore, threre is a possibility that the results of this study show more biased results due to selection bias for family physicians who are more active in family medicine society rather than the general characteristics of the entire family medicine specialists in Korea. In order to overcome this limitation, large scale studies will be necessary to conduct sophisticated research on representative samples population in the future. Nevertheless, this study is the first survey of family medicine specialists, as far as we know, that helps identify the medical practices of family medicine doctors and confirms the impact of decisions made during training on the medical care they will provide.

## Conclusion

More than 35 years have passed since family medicine has been recognized as a specialized department in Korea, and steady efforts have been made to implement primary care medicine based on initial medical contacts, accessibility, comprehensiveness, coordination, sustainability, and accountability [14]. However, there has been a lack of political support from the government to establish the proper function of primary medicine, and the position of the primary clinics has been gradually reduced in a system in which they compete with hospitals. The first step to prepare for the future is to identify the current status of family medicine physicians: What they are doing and thinking in the competitive medical situation in Korea. With a rapidly aging society, the importance of chronic disease management and comprehensive and continuous medical care is emphasized; Family medicine as a primary health care provider is eager to help patients navigate the challenging medical environment. This study is expected to be useful in establishing a clear direction for residency training, education programs, and for maintaining and developing the identity of family medicine. Large-scale follow-up studies using big data to facilitate comparison with other medical departments will be needed in the future.

## Supplementary information

**Additional file 1 Table S1.** The reasons that the chosen medical field is not covered by national insurance.

**Additional file 2 Figure S1** Distribution of working days during the week for family medicine physicians in clinics and hospitals.

## Data Availability

The datasets generated and analysed during the current study are not publicly available due to confidentiality, but are available from the corresponding author on reasonable request.

## Abbreviation

NHIS: National Health Insurance Service
