# Supplementary material for: A survey of the working status of family medicine physicians in clinics and hospitals in Korea
Source: BMC Fam Pract. 2020 May 8;21:82. doi: 10.1186/s12875-020-01154-5 (PMC7323610; doi:10.1186/s12875-020-01154-5)
Supplement: Supplementary file 1 — Additional file 1 Table S1. The reasons that the chosen medical field is not covered by national insurance. [file 12875_2020_1154_MOESM1_ESM.docx]

**Supplementary Table 1.** The reasons that the chosen medical field is not covered by national insurance.

|  |  | |
| --- | --- | --- |
| Total *N* = 422 | ***N*** | **%** |
| Economic reasons | 189 | 44.8 |
| Personal interest | 85 | 20.1 |
| No special reason | 82 | 19.4 |
| Medical independence from government | 44 | 10.4 |
| Others | 22 | 5.2 |
