# Supplementary figures and images for: A survey of the working status of family medicine physicians in clinics and hospitals in Korea
Source: BMC Fam Pract. 2020 May 8;21:82. doi: 10.1186/s12875-020-01154-5 (PMC7323610; doi:10.1186/s12875-020-01154-5)

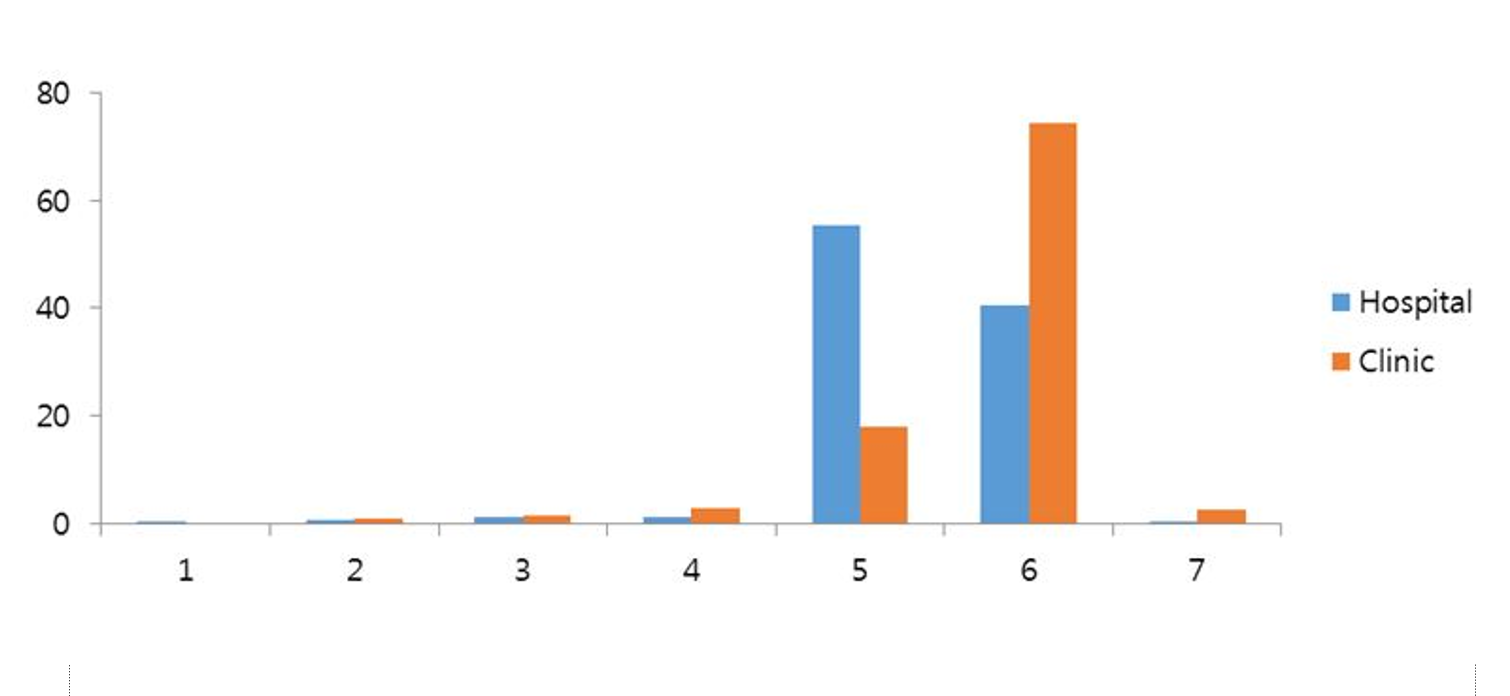

Supplement: Supplementary file 2 — Additional file 2 Figure S1 Distribution of working days during the week for family medicine physicians in clinics and hospitals. [file 12875_2020_1154_MOESM2_ESM.png]
